# Wood’s Library of Standard Medical Authors

**Published:** 1878-09-20

**Authors:** 


### WOOD'S LIBRARY OF STANDARD MEDICAL AUTHORS.

Messrs. Wm. Wood & Co. have announced that in January, 1879, they will begin the publication of Medical Books by the most distinguished modern and standard authors, in monthly volumes of from 200 to 300 pages and upwards, handsomely and strongly bound, at the merely nominal price of One Dollar each.

Estimating from the regular prices of the books so far selected for publication in 1879, subscribers to this Library will obtain about fifty dollars' worth of medical books for twelve dollars. This will furnish progressive medical men with a rare opportunity to procure new books at exceedingly low figures. Address Wm. Wood & Co., Book Sellers, N. Y.

